# A Predictive Model for Guillain–Barré Syndrome Based on Ensemble Methods

**DOI:** 10.1155/2018/1576927

**Published:** 2018-11-05

**Authors:** Juana Canul-Reich, José Hernández-Torruco, Oscar Chávez-Bosquez, Betania Hernández-Ocaña

**Affiliations:** División Académica de Informática y Sistemas, Universidad Juárez Autónoma de Tabasco, Tabasco, Mexico

## Abstract

Nowadays, Machine Learning methods have proven to be highly effective on the identification of various types of diseases, in the form of predictive models. Guillain–Barré syndrome (GBS) is a potentially fatal autoimmune neurological disorder that has barely been studied with computational techniques and few predictive models have been proposed. In a previous study, single classifiers were successfully used to build a predictive model. We believe that a predictive model is imperative to carry out adequate treatment in patients promptly. We designed three classification experiments: (1) using all four GBS subtypes, (2) One versus All (OVA), and (3) One versus One (OVO). These experiments use a real-world dataset with 129 instances and 16 relevant features. Besides, we compare five state-of-the-art ensemble methods against 15 single classifiers with 30 independent runs. Standard performance measures were used to obtain the best classifier in each experiment. Derived from the experiments, we conclude that Random Forest showed the best results in four GBS subtypes classification, no ensemble method stood out over the rest in OVA classification, and single classifiers outperformed ensemble methods in most cases in OVO classification. This study presents a novel predictive model for classification of four subtypes of Guillain–Barré syndrome. Our model identifies the best method for each classification case. We expect that our model could assist specialized physicians as a support tool and also could serve as a basis to improved models in the future.

## 1. Introduction

Guillain–Barré syndrome (GBS) is an autoimmune neurological disorder characterized by a fast evolution; usually, it goes from a few days up to four weeks, becoming the most common cause of acute paralysis of the peripheral nervous system in developed countries [1].

Complications of GBS vary among subtypes, which can be mainly acute inflammatory demyelinating polyneuropathy (AIDP), Acute motor axonal neuropathy (AMAN), Acute motor sensory axonal neuropathy (AMSAN), and Miller-Fisher syndrome (MF) [2, 3].

There are some works oriented to build a predictive model for this disorder using Machine Learning techniques with mechanical ventilation or respiratory failure as the dependent variable. These works consider clinical/physiological predictors mostly [4–8].

In this study, we investigate the predictive power of a reduced set of only 16 features selected out from an original dataset of 365 features. This dataset holds data from 129 Mexican patients and contains the four GBS subtypes mentioned above. We selected five ensemble methods: Boosting, Bagging, C5.0, Random Forest, and Random Subspace. In principle, ensemble learning combines multiple classifiers to obtain better predictive performance than that individually obtained from any of the constituent classifiers. These five methods were applied in three test scenarios, four GBS subtypes classification, One versus One (OVO) classification, and One versus All (OVA) classification, and compared their performance. In a previous study [9], we investigated the performance of 15 different-in-nature classifiers such as decision trees (C4.5), instance-based learners (*k*NN: *k* Nearest neighbor), kernel-based (SVM: Support vector machines), neural networks (SLP, MLP, RBF-DDA), and rule induction learners (OneR, JRip), among others. In this work, we made a performance comparison between single against ensemble classifiers.

There is evidence of previous work [10], where Uncini and collaborators conducted a study to increase the accuracy of electrodiagnostic criteria (using variables from nerve conduction studies) of the demyelinating and axonal subtypes of GBS. For this, they used sparse Linear Discriminant Analysis (LDA), two sets of existing electrodiagnostic criteria [11, 12], and one proposed by the authors to further evaluate the duration of motor responses and the sural preservation pattern and to define the reversible conduction failure (RCF) in the motor and sensory nerves in a second study.

The misclassification error rates at their first study, compared to reference diagnoses, were 15.3% for sparse LDA, 30% for our criteria, 45% for Rajabally's, and 48% for Hadden's. Sparse LDA identified seven most relevant electrophysiological variables in differentiating demyelinating and axonal subtypes. With this, they assigned to each patient the diagnostic probability of belonging to either subtype.

The authors found that the signs of the coefficients of variables indicated that AIDP, as compared to axonal GBS, is characterized by higher values of peroneal DML (Distal motor latency), ulnar dCMAP duration (distal Compound motor action potential), ulnar and median proximal/distal (p/d) CMAP amplitude ratio, and lower median Sensory Nerve Action Potential (SNAP) amplitude, as well as lower peroneal Motor conduction velocity (MCV) and lower ulnar SNAP/sural SNAP amplitude ratio.

Uncini et al. focus only on classifying AIDP against axonal (AMAN and AMSAN) subtypes. However, in this study, we go further by conducting experiments for the classification of all four common GBS subtypes: AIDP, AMAN, AMSAN, and MF. Moreover, we performed experiments in three scenarios: using four GBS subtypes at the same time, OVA, and OVO. For this study, we used 16 relevant features. Also, an experiment was performed using the 156 features with the aim to analyze the effect of using only the 16 relevant features in the classification tasks.

This study contributes to the effort in creating a predictive model for GBS subtype classification. Also, the analysis performed in this work provides insight into the best single classifiers for each classification case. Further experiments with additional algorithms are in the schedule.

This paper is organized as follows: Section 2 outlines the materials used and the methods applied.  Section 3 describes the experimental results. In Section 4, we discuss the results. Finally, in Section 5, general conclusions of this study are presented, and we also suggest future works.

## 2. Materials and Methods

In this section, details of the dataset used in the experiments, the description of the metrics used for performance evaluation, and the report of the tested classifiers are given, as well as the experimental design conducted.

### 2.1. Data

Dataset comes from the Instituto Nacional de Neurología y Neurocirugía, located in Mexico City, with records of 129 patients already classified as one of the GBS subtypes:

- 20 AIDP
- 37 AMAN
- 59 AMSAN
- 13 Miller-Fisher

The original dataset contains 365 features. From these, we detected a subset of 16 relevant ones in a previous study [4]:

- v22: symmetry (in weakness)
- v29: extraocular muscles involvement
- v30: ptosis
- v31: cerebellar involvement
- v63: amplitude of left median motor nerve
- v106: area under the curve of left ulnar motor nerve
- v120: area under the curve of right ulnar motor nerve
- v130: amplitude of left tibial motor nerve
- v141: amplitude of right tibial motor nerve
- v161: area under the curve of right peroneal motor nerve
- v172: amplitude of left median sensory nerve
- v177: amplitude of right median sensory nerve
- v178: area under the curve of right median sensory nerve
- v186: latency of right ulnar sensory nerve
- v187: amplitude of right ulnar sensory nerve
- v198: area under the curve of right sural sensory nerve

In summary, four features are clinical while the 12 remaining features were obtained from a nerve conduction test.

We use the following method to identify these 16 relevant features:

1. Preselect certain variables using the diagnostic criteria for GBS according to the literature. The resulting dataset contains 156 variables: 121 variables from the nerve conduction test, four variables from the CSF analysis, and 31 clinical variables.
2. We proposed a novel method combining quenching simulated annealing [9] (QSA) and Partitions around medoids (PAM) (the QSA-PAM method). QSA is a metaheuristic that generates approximate-to-the-optimal solutions in reasonable times for complex combinatorial problems. We applied QSA to select different random feature subsets from the dataset. These new datasets created using feature subsets served as input to PAM to build four clusters.
3. A clustering technique was needed because this method is useful to unveil the existence of groups of homogeneous data. As we knew in advance the presence of four GBS classes in the dataset, we could straightforwardly identify the relevant features to build four clusters, each corresponding to a GBS subtype.
4. Purity, a quality metric, was used to check each cluster's quality. Groups with the highest purity contain the most significant number of elements of the same type and the fewest number of items of a different type.
5. We measure the purity of clusters concluding that 16 features from the original dataset were relevant for identifying GBS subtypes (highest purity = 0.8992).

### 2.2. Ensemble Methods

In this study, we include results from five ensemble methods. Following this, we show the ensemble methods with their parameters:

1. Boosting: boosting iterations
2. Bagging: number of trees
3. C5.0: number of trials
4. Random Forest: number of variables in the random subset at each node and number of trees in the forest
5. Random Subspace: subspace sample size and number of iterations

We compare results from these ensembles against those obtained by single classifiers from a previous study [9]. Previously, we showed promising ensemble results by combining trees using C5 and Random Forest only [13]. We further investigated the performance of an ensemble approach with additional combination methods such as Random Subspace, Bagging, and Boosting. Thorough ensemble results are given in this work. The complete list of single classifiers is given in Table 1.

#### 2.2.1. Boosting

It is a type of ensemble method that combines multiple homogeneous classifiers by voting [14]. Boosting aims at turning a set of weak learners into a strong learner. A weak learner is a classifier that slightly correlates with the true classification (it just can label examples better than random guessing). In contrast, a strong learner is a classifier that is arbitrarily well-correlated with the true classification.

Boosting is iteratively applied to the data so that a sequence of weak classifiers is produced. Boosting assigns weights to every instance. Initially, all instances have the same weight. At each iteration, the weights are modified by increasing the weights of the misclassified instances to have the weak learners focus more on these. As iterations go by, less misclassified instances are obtained. Finally, all the weak classifiers are combined by weighted voting where the weight assigned to each classifier depends on its error rate. In this work, we implemented the AdaBoost (Adaptive Boosting algorithm) [15], which uses decision trees as weak learners.

#### 2.2.2. Bagging

Introduced by Leo Breiman, its meaning is bootstrap aggregating. Bagging is a method for generating multiple versions of a predictor and using these to get an aggregated predictor [16]. Bagging generates *m* new training sets by making bootstrap replicates from the original training set. The *m* models are trained using a base classifier with these *m* bootstrap (random sampling with replacement) samples. Then, each resultant model predicts a test set. All predictions are combined by averaging the output (for regression) or voting (for classification). In this work, Bagging was implemented using decision trees as single classifiers.

#### 2.2.3. C5.0

Introduced by Ross Quinlan [17], it is an improved version of C4.5. Its significant improvement is the implementation of Boosting which enhances trees and gives them higher precision. The differences between the algorithm used in C5.0 and AdaBoost are the following [18]: (1) C5.0 tries to maintain a tree size similar to the initial one (which is generated without Boosting being involved). This is correlated with the number of terminal nodes, which increase in number as the tree grows. (2) C5.0 calculates class probabilities for all boosted models, and within these models, weighted averages are calculated. Then, from these models, C5.0 chooses the class having the maximum probability within the group.

#### 2.2.4. Random Forest

It was introduced by Breiman and Adele Cutler [19] and is a predictive algorithm built by a bootstrap ensemble of CART trees. Given *N* number of training data points and *M* number of predictor variables, this algorithm generates many bootstrap samples by selecting *N* data points with replacement from the training dataset. Then, a CART tree is trained on each bootstrap sample using *m* randomly chosen predictors out of the original *M* predictors (*m* << *M* if *M* is large). The trees are fully grown without pruning. Random Forest is robust against overfitting.

#### 2.2.5. Random Subspace

It was introduced by Tim Ho [20] and consists of several base classifiers each operating in randomly chosen subspaces of the original feature space. These classifiers are usually combined by simple majority voting to generate the final class.

### 2.3. Performance Measures

We apply standard performance measures as accuracy, balanced accuracy, sensitivity, and specificity, along with the Kappa Statistic.

#### 2.3.1. Accuracy

It is the most typical performance metric used in classification. It is the ratio of correctly classified instances to the total number of instances in the dataset.

#### 2.3.2. Balanced Accuracy

It is a classification performance metric conveniently applied when imbalanced datasets are used in experiments. It is defined as

(1) $$\begin{array}{c} \text{balanced accuracy}=\frac{\left(\text{TP}/\left(\text{TP}+\text{FN}\right)\right)+\left(\text{TN}/\left(\text{FP}+\text{TN}\right)\right)}{2}, \end{array}$$

where TP = true positive, FN = false negative, TN = true negative, and FP = false positive.

#### 2.3.3. Sensitivity

It indicates the goodness of a classifier to classify true positives. That is, in a diagnostic test, it would be the ability to classify ill people accurately. It is defined as

(2) $$\begin{array}{c} \text{sensitivity}=\frac{\text{TP}}{\text{TP}+\text{FN}}. \end{array}$$

#### 2.3.4. Specificity

It indicates the goodness of a classifier to identify true negatives. That is, in a diagnostic test, it would be the ability to classify healthy people accurately. It is defined as

(3) $$\begin{array}{c} \text{specificity}=\frac{\text{TN}}{\text{TN}+\text{FP}}. \end{array}$$

#### 2.3.5. Kappa Statistic

Introduced by [21], it measures the agreement between predicted versus ground truth classifications of a dataset. At the same time, it corrects randomly occurred agreement [14].

According to [22], the Kappa statistic lies in the range from 0 to 1 as follows:

  0 = agreement equivalent to chance
  0.1 0.20 = slight agreement
  0.21 0.40 = fair agreement
  0.41 0.60 = moderate agreement
  0.61 0.80 = substantial agreement
  0.81 0.99 = near perfect agreement
  1 = perfect agreement

We applied standard performance measures in Machine Learning such as sensitivity, specificity, error rate, ROC curves, and Kappa statistic. Also, we included average accuracy and balanced accuracy. The former is used in four GBS subtype classification, since it is a more suitable measure for multiclass classification problems. The latter is used in OVA and OVO classification, because it is a better performance estimate of imbalanced datasets.

Accuracy is the typical performance measure used in classification representing the number of correct classifications. For example, an accuracy of 0.9 means a 90% of correct classifications.

### 2.4. Experimental Design

We used the 16-feature subset, described in Section 2.1, for experiments. We added the class variable to this subset, that is, the GBS subtype. Finally, we created a dataset containing the 129 instances and 17 features. As mentioned in Section 2.1, our dataset has four classes, identified with numbers 1 to 4, where 1 = AIDP, 2 = AMAN, 3 = AMSAN, and 4 = MF.

We employed a stratified train-test evaluation scheme in all cases, two-thirds of data for training, and one-third for testing. We performed 30 runs where we applied each of the methods described in Section 2.2. In each run, we set a different seed. Same seeds were used for each classifier. These seeds were generated using Mersenne-Twister pseudo-random number generator [23]. The use of a different seed for each run ensures different splits of train and test sets.

The base classifier in Random Subspace method used was the best single classifier for each case using train-test, and the complete list is in Table 2. Experiments of Random Subspace were performed in Weka 3.6.12. SVMLap is not implemented in Weka 3.6.12 [14]. Therefore, we used SVMPoly (second best) [9] instead of AIDP versus AMSAN classification.

#### 2.4.1. Four GBS Subtypes Classification

In this classification scenario, the four GBS subtypes were included in the dataset, that is, AIDP, AMAN, AMSAN, and MF. In this scenario, the base metric was the average accuracy.

#### 2.4.2. OVA Classification

For OVA classification scenario, we created four new datasets, as many as the number of GBS subtypes in the dataset. In each one, instances of one class were marked as the positive cases, and instances of the remaining classes (labeled as ALL) were marked as the negative cases. In this scenario, the base metric was the balanced accuracy.

#### 2.4.3. OVO Classification

For OVO classification scenario, we created six new datasets, as many as the number of combinations of pairs of GBS subtypes. Each dataset contained instances of only two GBS subtypes, one class marked as the positive case and the other class as the negative case. In this scenario, the base metric was the balanced accuracy.

#### 2.4.4. Train-Test

For each run, we computed accuracy, sensitivity, specificity, Kappa statistic, and multiclass AUC. Finally, we averaged each of these quantities across the 30 runs.

#### 2.4.5. Parameter Optimization/Setting

Parameter optimization for all classifiers was performed using the dataset with four GBS subtypes. Figures and tables are shown in Supplementary Material.

- Boosting. The number of boosting iterations was optimized by performing 30 train-test runs for each value from 10 to 100. The highest average accuracy across 30 runs was found with a number of iterations equal to 50, as shown in Table 4. This value was used for all experiments with Boosting including four SGB subtypes, OVA and OVO classifications.
- Bagging. The optimal number of trees used for all cases was 100. This number was found by performing 30 train-test runs where the average accuracy was calculated for each value from 10 to 100.  Table 5 shows the values found for each number of trees. This value was used for all experiments with Bagging including four SGB subtypes, OVA, and OVO classifications.
- C5.0. It requires the optimization of the number of trials. The tuning of this parameter was performed by the training-test runs using different numbers of trials ranging from 5 to 100.
- Figure 2 shows the results of C5.0 optimization. The lowest average error rate across the train-test runs was obtained with a number of trials = 55. Experiments for all cases in C5.0, including OVO and OVA classification, were performed using this number of trials.
- Random Forest. This method has only two tuning parameters: the number of variables in the random subset at each node and the number of trees in the forest. In this work, we use a Random Forest implementation in *R* language [24] which automatically tune the first parameter. In order to tune the second parameter, we performed 30 training-test runs using different numbers of trees from 100 to 1000.
- Figure 3 shows the results of Random Forest optimization. The lowest average error rate across the train-test runs was obtained with the number of trees = 700. Experiments for all cases in Random Forest, including OVO and OVA classification, were performed using this number of trees.
- Random Subspace. In this work, the subspace sample size was set to 0.25, meaning that for each model, only 25% of the features are randomly selected. The number of iterations for Random Subspace was set to 50. These measures were obtained from a tuning phase where different values for subspace sample size and number of iterations were tried in 30 train-test runs.  Table 6 shows the complete tuning results. As for the base classifiers, the same optimal parameter setting obtained in previous single classification experiments [9] was used in this study. Table 2 shows the complete list of base classifiers configuration.

## 3. Results

This section presents the results of each of the ensemble classifiers in all three experiments: *(i)* all subtypes (AIDP. AMAN, AMSAN, MF), *(ii)* OVO (AIDP vs. AMAN, AIDP vs. AMSAN, AIDP vs. MF, AMAN vs. AMSAN, AMAN vs. MF, AMSAN vs. MF), and *(iii)* OVA (AIDP vs. ALL, AMAN vs. ALL, AMSAN vs. ALL, MF vs. ALL). The performance of combined classifiers is compared with that of simple classifiers.

### 3.1. Four GBS Subtypes Classification

In this section, we show the results of ensemble methods in four GBS subtypes classification.  Table 3 shows the average results across all runs along with the standard deviation (sd). Four of the five ensemble methods obtained an average accuracy above 0.90. Random Forest outperformed the rest of the methods in most of the metrics. The worst performance was shown by Bagging, with an average accuracy of 0.89 along with poor results in sensitivity and Kappa statistic.

Multiclass AUC ranged in 0.78–0.83. Specificity values were higher than those of sensitivity. Specificity ranged in 0.92–0.95, while sensitivity ranged in 0.66–0.81. Kappa ranged in 0.69–0.80. Overall, four GBS subtypes classification using ensemble methods obtained high values in average accuracy. The remaining metrics showed a large variation.

**Figure 1:**
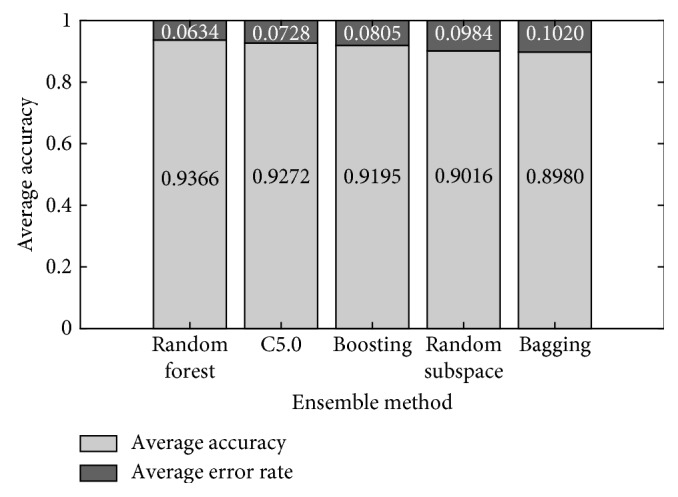
Average accuracy and average error rate of ensemble methods across 30 runs in four GBS subtype classification.

Figure 1 shows the average accuracy across the runs for each ensemble method in four GBS subtypes classification. Also, the average error rate for each method is shown. Most of the methods obtained an average accuracy above 0.90. Random Forest obtained the lowest average error rate across all train-test runs.

Table 7 in Supplementary Material shows the average accuracy of single classifiers and ensemble methods across the runs of four GBS subtypes classification. Only two ensemble methods, Random Forest and C5.0, outperformed all single classifiers in average accuracy. Boosting resulted better than 13 of 14 single classifiers. Random Subspace had performance comparable to that of SVMLin and Naive Bayes. However, Random Subspace failed at improving *k*NN as a single classifier. As shown in Table 7, *k*NN, when used as single classifier, obtained a higher average accuracy (0.9268) than that when used in Random Subspace as a base classifier (0.9016). The worst performance of ensemble methods was shown by Bagging; however, it was better than half of the single classifiers.

### 3.2. Impact Analysis of the 16 Relevant Features in the Diagnostic Model

We conducted the same experimental design described above in Section 2.4 using the original 156 variables with both single classifiers and ensemble methods. This experiment was carried out with the objective of analyzing the impact of the feature selection process where a subset of 16 relevant features was determined as described in Section 2.1. The experiment was carried out using the four GBS subtypes present in the dataset.

Using the single classifiers, we found an absolute difference in the average accuracy in the range of 0.0049 to 0.2151. This difference is using the 156 variables and the 16 relevant variables identified in the process described previously. The single classifier with the biggest difference was RBF-DDA, with a difference of 0.2151. The least affected single classifier was linear SVM with 0.0049. See Tables 8 and 9 for details (in Supplementary Material).

Using the ensemble methods, we found an absolute difference in the average accuracy in the range of 0.0001 to 0.1853. The ensemble method with the biggest difference was Random Subspace, with a difference of 0.1853. The least affected single classifier was Bagging with 0.0001.

In all cases, results are better using the 16 relevant features. These hold true for both the single classifiers and the ensemble methods.

### 3.3. OVA Classification

In this section, we show the results of ensemble methods across the runs in OVA classification, that is, AIDP versus ALL, AMAN versus ALL, and so on (tables are shown in Supplementary Material).  Table 10 shows the average results of ensemble methods across all runs along with the standard deviation (sd) in AIDP versus ALL classification. Only two of the five ensemble methods obtained a balanced accuracy above 0.80, and these were C5.0 and Boosting. The worst performance was obtained by Random Subspace, with a balanced accuracy of 0.68 and poor results in most metrics. However, Random Subspace obtained an unusual high specificity value. This means that classifiers were more able to identify instances from all other GBS subtypes (ALL) than those belonging to AIDP.

AUC ranged in 0.68–0.81. Specificity was higher than sensitivity. Specificity ranged in 0.92–0.99, while sensitivity ranged in 0.36–0.69. Kappa ranged in 0.46–0.64. In summary, ensemble methods obtained a low performance in most metrics in AIDP versus ALL classification.

Table 11 shows the balanced accuracy of single classifiers and ensemble methods across the runs in AIDP versus ALL classification. No ensemble method was able to improve *k*NN, a single classifier, in balanced accuracy. Only two ensemble methods, C5.0 and Boosting, outperformed the rest of single classifiers in balanced accuracy. Random Subspace had a poor performance, only being better than the worst single classifier, OneR. Again, Random Subspace was not able to outperform *k*NN as a single classifier.

Table 12 shows the average results of ensemble methods across the runs in AMAN versus ALL classification. The standard deviation is also shown. Four of the five ensemble methods obtained a balanced accuracy above 0.90, only Bagging was under this value. AUC ranged in 0.85–0.92. Values obtained in specificity were higher than those obtained in sensitivity. Specificity ranged in 0.92–0.94, while sensitivity ranged in 0.78–0.91. Kappa ranged in 0.73–0.83. In short, ensemble methods obtained values on or above 0.85 in most metrics in AMAN versus ALL classification.

Table 13 shows the balanced accuracy of single classifiers and ensemble methods across the runs in AMAN versus ALL classification. Two single classifiers outperformed all the ensemble methods, *k*NN and SVMGaus. Boosting resulted better than 12 single classifiers and four ensemble methods. Like in the former cases, Random Subspace was not able to outperform *k*NN as a single classifier. Bagging was the worst ensemble method in AMAN versus ALL classification.

Table 14 shows the average results of ensemble methods across the runs in AMSAN versus ALL classification. The standard deviation is also shown. All five ensemble methods obtained a balanced accuracy above 0.85. AUC ranged in 0.85–0.89. Like all other cases, specificity was higher than sensitivity. Specificity ranged in 0.87–0.93, while sensitivity ranged in 0.83–0.86. Kappa ranged in 0.71–0.78. Overall, ensemble methods had a high performance in AMSAN versus ALL classification in most metrics.

Table 15 shows the balanced accuracy of single classifiers and ensemble methods across the runs in AMSAN versus ALL classification. Random Forest was the best ensemble method with a balanced accuracy of 0.8924. However, it was not able to outperform *k*NN, the best single classifier with 0.8953 of balanced accuracy. C4.5, a single classifier, was the third best method, and it had a higher balanced accuracy than four ensemble methods and 12 single classifiers. Neither in this case was Random Subspace able to improve *k*NN as a single classifier.

Table 16 shows the average results of ensemble methods across the runs in MF versus ALL classification. The standard deviation is also shown. Three ensemble methods obtained a balanced accuracy above 0.80. AUC ranged in 0.74–0.85. Sensitivity was much lower than specificity than that in previous cases. Sensitivity ranged in 0.52–0.76. Specificity ranged in 0.90–0.96. Kappa ranged in 0.49–0.64. In summary, ensemble methods had a poor performance in MF versus ALL classification in most metrics.

Table 17 shows the balanced accuracy of single classifiers and ensemble methods across the runs in MF versus ALL classification. Naive Bayes, a single classifier, obtained the highest balanced accuracy outperforming all ensemble methods. Random Subspace was the best ensemble method. However, it was not able to improve Naive Bayes as a single classifier. Three ensemble methods were better than most of single classifiers, and these were Random Subspace, Bagging, and C5.0.

### 3.4. OVO Classification

Regarding the results of ensemble methods in OVO classification, tables are shown in Supplementary material.

Table 18 shows the average results of ensemble methods across the runs in AIDP versus AMAN classification. The standard deviation is also shown. All ensemble methods obtained a balanced accuracy above 0.90. Also, AUC surpassed this value. Values obtained in sensitivity were lower than those obtained in specificity. Sensitivity ranged in 0.84–0.95. Specificity ranged in 0.96–0.97. Kappa ranged in 0.83–0.91. Overall, ensemble methods obtained values on or above 0.90 in most metrics in AIDP versus AMAN classification.

Table 19 shows the balanced accuracy of single classifiers and ensemble methods across the runs in AIDP versus AMAN classification. JRip slightly outperformed Bagging and C5.0, as the best classifier in this case. Two rule induction learners, JRip and OneR, were at the top four classifiers in AIDP versus AMAN classification. JRip as a single classifier was not outperformed by Random Subspace when used as the base classifier.

Table 20 shows the average results of ensemble methods across the runs in AIDP versus AMSAN classification. The standard deviation is also shown. Random Forest obtained a balanced accuracy above 0.90, and the rest of the ensemble methods went above 0.85. Values obtained in sensitivity were lower than those obtained in specificity. Sensitivity ranged in 0.78–0.85. Specificity ranged in 0.90–0.96. Kappa ranged in 0.70–0.83. In short, ensemble methods had a high performance in AIDP versus AMSAN classification in most metrics.

Table 21 shows the balanced accuracy of single classifiers and ensemble methods across the runs in AIDP versus AMSAN classification.

Random Forest obtained the highest balanced accuracy. The second best ensemble method was Boosting, only under single classifiers SVMLap and SVMPoly. In this case, SVMGaus was implemented as the base classifier in Random Subspace instead of SVMLap, as mentioned in Section 2.4. As in previous cases, Random Subspace did not outperform SVMGaus, its base classifier.

Table 22 shows the average results of ensemble methods across the runs in AIDP versus MF classification. The standard deviation is also shown. Random Subspace and Bagging obtained a balanced accuracy above 0.85, the rest of ensemble methods ranged in 0.76–0.83. Sensitivity was lower than specificity. Sensitivity ranged in 0.71–0.83. Specificity ranged in 0.82–0.99. Kappa ranged in 0.53–0.75. In summary, only Random Subspace and Bagging showed the best performance in AIDP versus MF classification in most metrics. The rest of ensemble methods had low performance.

Table 23 shows the balanced accuracy of single classifiers and ensemble methods across the runs in AIDP versus MF classification. Two ensemble methods, Random Subspace and Bagging, outperformed all the other methods, including single classifiers. In this case, Random Subspace was able to improve the performance of its base classifier, OneR.

Table 24 shows the average results of ensemble methods across the runs in AMAN versus AMSAN classification. The standard deviation is also shown. All ensemble methods obtained a balanced accuracy of around 0.90 and above. As in previous cases, sensitivity was lower than specificity. Sensitivity ranged in 0.88–0.93. Specificity ranged in 0.90–0.97. Kappa ranged in 0.78–0.89. Overall, ensemble methods obtained values on or above 0.85 in most metrics in AMAN versus AMSAN classification.

Table 25 shows the balanced accuracy of single classifiers and ensemble methods across the runs in AMAN versus AMSAN classification. *k*NN was the best classifier followed by Random Forest. In this case, Random Subspace was not able to improve the performance of its base classifier, *k*NN.

Table 26 shows the average results of ensemble methods across the runs in AMAN versus MF classification. The standard deviation is also shown. Four of five ensemble methods obtained a balanced accuracy above 0.90, and only Random Subspace had a poor result. In this case, being AMAN the majority class, sensitivity was higher than specificity. Sensitivity ranged in 0.95–0.99. Specificity ranged in 0.50–0.87. Kappa showed a large variation, ranging from 0.57–0.89. Shortly, almost all ensemble methods obtained a remarkable performance in AMAN versus MF classification in most metrics.

Table 27 shows the balanced accuracy of single classifiers and ensemble methods across the runs in AMAN versus MF classification. Half of single classifiers outperformed ensemble methods, even though these last obtained a high performance. Random Subspace was the worst method, including single classifiers and ensemble methods.

Table 28 shows the average results of ensemble methods across the runs in AMSAN versus MF classification. The standard deviation is also shown. Four of five ensemble methods obtained a balanced accuracy above 0.85. Like the previous case, sensitivity was higher than specificity, because of the majority class effect. Sensitivity ranged in 0.89–0.95. Specificity ranged in 0.71–0.87. Low values were obtained in Kappa, which ranged from 0.65–0.71. Overall, almost all ensemble methods obtained values on or above 0.85 in most metrics in AMSAN versus MF classification.

Table 29 shows the balanced accuracy of single classifiers and ensemble methods across the runs in AMSAN versus MF classification. Naive Bayes resulted better than all ensemble methods and the rest of single classifiers. Random Subspace was the second best, and it almost reaches Naive Bayes performance, its base classifier.

## 4. Discussion

Our objective in this work was to investigate if ensemble methods were able to improve single classifiers in building a predictive model for GBS. We used the 16 relevant features identified with the QSA-PAM method as predictors. Also, we applied five ensemble methods: Boosting, Bagging, C5.0, Random Forest, and Random Subspace. We conducted three types of experiments: four GBS subtypes classification, OVA classification, and OVO classification. We compared the performance of both single classifiers and ensemble methods.

Many studies report significant differences in the severity and outcome of patients among the different subtypes of GBS [2, 25–30]. On the other hand, OVA and OVO are two approaches in Machine Learning to address the problem of multiclassification [31, 32]. These approaches are widely used in the diagnosis of multiple subtypes in other conditions [33–35]. These experiments provide insight into how well one subtype distinguishes from another and also how well one subtype distinguishes against the others. Also, from Machine Learning perspective, it is interesting to analyze which of the two approaches is better in a particular disease and which classifier performs the best differentiation.

### 4.1. Four GBS Subtypes Classification

All ensemble methods accomplished well on the performance measures, where Random Forest and C5.0 had the best results in average accuracy, multiclass auc, sensitivity, specificity, and Kappa statistics with better quality according the standard deviation.

Two ensemble methods succeeded at improving the average accuracy of all single classifiers: Random Forest and C5.0. Random Forest surpassed *k*NN by almost a percentage unit. C5.0 barely made it.

### 4.2. Impact Analysis of the 16 Relevant Features in the Diagnostic Model

Regardless of results found in these experiments, the fact of having a simple diagnostic model for the subtypes of GBS that uses only 16 relevant features represents a contribution because it allows directly performing GBS subtypes differentiation. It describes an advantage from the medical point of view, and thus for physicians, the diagnostic process is eased by using a smaller number of variables. Moreover, from the Machine Learning perspective, the efficiency of the feature selection methods was as expected.

### 4.3. OVA Classification

The best results were obtained in AMAN versus ALL, followed by AMSAN versus ALL, in both cases with a balanced accuracy of over 0.85. The worst classification was obtained in AIDP versus ALL with a Balanced accuracy lower than 0.82.

In all four cases, different classifiers obtained the best performance: in AIDP versus ALL was C5.0, in AMAN versus ALL was Boosting, in AMSAN versus ALL was Random Forest, and in MF versus ALL was Random Subspace. No ensemble method stood out over the rest.

In regards to single classifiers, *k*NN outperformed all methods in three cases, followed by Naive Bayes in one case.

### 4.4. OVO Classification

The best results were obtained in classifications with the AMAN class: AIDP versus AMAN, AMAN versus AMSAN, and AMAN versus MF. Random Forest was the best classifier in three cases: AIDP versus AMSAN, AMAN versus AMSAN, and AMAN versus MF. Random Subspace was the best classifier in two cases: AIDP versus MF and AMSAN versus MF.

In AIDP versus MF and AIDP versus AMSAN classifications, ensemble methods outperformed single classifiers: Random Subspace in the first case and Random Forest in the second.

*k*NN was the best classifier in four cases, including single classifiers and ensemble methods.

Single classifiers outperform ensemble methods in most cases. Three cases were the exception: four GBS subtypes classification, AIDP versus AMSAN, and AIDP versus MF. This result requires further investigation.

## 5. Conclusions

In this work, we aimed at creating the highest accurate predictive model for GBS possible, using the 16 relevant features identified with the QSA-PAM method. This effort enriches our previous work on this topic using Machine Learning methods. For this approach, we applied five ensemble methods: Boosting, Bagging, C5.0, Random Forest, and Random Subspace. We compare the results obtained by these methods against previous results using 15 single classifiers: *k*NN, SVMLin, SVMPoly, SVMGaus, SVMLap, C4.5, SLP, MLP, RBF-ANN, JRip, OneR, Naive Bayes, BLR, MLR, and LDA.

Three types of experiments were performed: four GBS subtypes classification, OVA classification, and OVO classification in order to make the comparison.

In the first experiment, Random Forest was the best ensemble method and outperformed all single classifiers.

In the second experiment, no ensemble method stood out over the rest in all four classifications. However, single classifiers outperformed ensemble methods in all cases.

Finally, in the last experiment, Random Subspace and Random Forest were the best ensemble methods. Also, these methods outperformed single classifiers in two classifications.

We consider that the proposed predictive model identifies the best method for each classification case. Knowing which classifiers are the best in the diagnostic tasks in the different scenarios (4 subtypes, OvO, and OvA) could serve as a basis to build an expert system that implements the best models. This system would facilitate the decision making of physicians in the diagnosis of subtypes. As we mentioned before, many studies report significant differences in the severity and outcome of patients among the different subtypes of GBS. Knowing in advance the specific subtype of GBS suffered by the patient allows the physicians and patient's relatives to take the appropriate measures for their recovery.

A priori, ensemble methods are expected to outperform single classifiers, due to the reason that they use different strategies designed for this purpose, usually consisting of repeating the classification process with the misclassified examples by giving them greater weight in future iterations (Boosting) and until using multiple classification trees in combination with methods of sampling with replacement (Random Forest). In this study, we make this analysis with five different ensemble methods and 15 single classifiers. It represents a contribution in the Machine Learning area. From Neurology perspective, this contribution consists of indicating which of the single classifiers and the ensemble methods are the best in the tasks of distinguishing between subtypes of the SGB.

As future work, we will further tackle the imbalanced data problem. We are also interested in investigating the optimal tuning of the parameters used in Boosting, Bagging, and Random Subspace. The models generated by the classifiers mentioned above can be embedded in expert systems to act as assistants in the decision making of the specialists.

## Figures and Tables

**Table 1:** List of single classifiers used in our previous study [13]. Binary Logistic Regression (BLR) used in OVA and OVO classifications. Multinomial Logistic Regression (MLR) used in four GBS subtype classification.

Single classifier	Approach	Tuning parameter
*k*NN	Instance-based	*k*, *d*
SVM linear kernel (SVMLin)	Kernel-based	*C*
SVM polynomial kernel (SVMPoly)	Kernel-based	*C*, degree, *σ* (*γ*), coef
SVM Gaussian kernel (SVMGaus)	Kernel-based	*C*, *σ* (*γ*)
SVM laplacian kernel (SVMLap)	Kernel-based	*C*, *σ* (*γ*)
C4.5	Decision tree	NA
Single layer perceptron (SLP)	Neural network	Size, decay
Multilayer perceptron (MLP)	Neural network	Size
Radial basis function ANN (RBF-ANN)	Neural network	Negative threshold
JRip	Rule induction	NumOpt
OneR	Rule induction	NA
Naive bayes	Bayesian	NA
Binary logistic regression (BLR)	Regression	NA
Multinomial logistic regression (MLR)	Regression	NA
Linear discriminant analysis (LDA)	Discriminant analysis	NA

**Table 2:** Base classifiers used in Random Subspace for each classification case.

Base classifier	Parameter setting	Classes
*k*NN	*k* = 18, *d* = Manhattan	AIDP, AMAN, AMSAN, MF
*k*NN	*k* = 18, *d* = Manhattan	AIDP vs. ALL
*k*NN	*k* = 18, *d* = Manhattan	AMAN vs. ALL
*k*NN	*k* = 18, *d* = Manhattan	AMSAN vs. ALL
Naive bayes	—	MF vs. ALL
yjJRip	NumOpt = 3	AIDP vs. AMAN
SVMGaus	*s* = 0.01, *C* = 10	AIDP vs. AMSAN
OneR	—	AIDP vs. MF
*k*NN	*k* = 18, *d* = Manhattan	AMAN vs. AMSAN
SVMGaus	*s* = 0.01, *C* = 10	AMAN vs. MF
Naive bayes	—	AMSAN vs. MF

**Table 3:** Average results of ensemble methods across 30 runs in four GBS subtype classification.

Ensemble method	Average accuracy	Multiclass AUC	Sensitivity	Specificity	Kappa
**Random Forest**	**0.9366**	**0.8390**	**0.8120**	**0.9544**	**0.8090**
	0.0245	0.0803	0.0812	0.0178	0.0748

**C5.0**	**0.9272**	**0.8398**	**0.8126**	**0.9476**	**0.7825**
	0.0251	0.0789	0.0749	0.0191	0.0746

**Boosting**	**0.9195**	**0.8099**	**0.7906**	**0.9422**	**0.7596**
	0.0202	0.0578	0.0648	0.0158	0.0610

**Random Subspace**	**0.9016**	**0.7871**	**0.6607**	**0.9251**	**0.6960**
	0.0216	0.0592	0.0691	0.0169	0.0682

**Bagging**	**0.8980**	**0.7895**	**0.6936**	**0.9251**	**0.6923**
	0.0284	0.0484	0.0622	0.0206	0.0831

## Data Availability

The data used to support the findings of this study are available from the corresponding author upon request.

## Supplementary Materials

Supplementary Materials We provide supplementary material organized in two sections: Experimental Design and Results. In the first section, we show results from Boosting (Table 4), Bagging (Table 5), C5.0 (Figure 2), Random Forest (Figure 3), and Random Subspace (Table 6) tuning procedures, as a complement for Parameter Optimization/Setting (Section 2.4.5). In the second section, Table 7 is complementary material for Four GBS Subtypes Classification (Section 3.1), Tables 8 and 9 are complementary material for Impact Analysis of the 16 Relevant Features in the Diagnostic Model (Section 3.2), Tables 10–17 are complementary material for OVA Classification (Section 3.3), and Tables 18–29 are complementary material for OVO Classification (Section 3.4).
