# Supplementary material for: A Predictive Model for Guillain–Barré Syndrome Based on Ensemble Methods
Source: Comput Intell Neurosci. 2018 Nov 5;2018:1576927. doi: 10.1155/2018/1576927 (PMC6247730; doi:10.1155/2018/1576927)
Supplement: Supplementary Materials — We provide supplementary material organized in two sections: Experimental Design and Results. In the first section, we show results from Boosting (Table 4), Bagging (Table 5), C5.0 (Figure 2), Random Forest (Figure 3), and Random Subspace (Table 6) tuning procedures, as a complement for Parameter Optimization/Setting (Section 2.4.5). In the second section, Table 7 is complementary material for Four GBS Subtypes Classification (Section 3.1), Tables 8 and 9 are complementary material for Impact Analysis of the 16 Relevant Features in the Diagnostic Model (Section 3.2), Tables 10–17 are complementary material for OVA Classification (Section 3.3), and Tables 18–29 are complementary material for OVO Classification (Section 3.4). [file 1576927.f1.pdf]

Supplementary material

1. Experimental design

Table 4: Tuning the number of iterations for Boosting.

Number of iterations	Average accuracy
10	0.9126
20	0.9159
30	0.9171
40	0.9181
50	**0.9195**
60	0.9189
70	0.9181
80	0.9177
90	0.9165
100	0.9154

Table 5: Tuning the number of trees for Bagging.

Number of trees	Average accuracy
10	0.8926
20	0.8935
30	0.8942
40	0.8951
50	0.8949
60	0.8953
70	0.8968
80	0.8972
90	0.8977
100	**0.8980**

Table 6: Random subspace parameter tuning.

	Subspace sample size		
Number of iterations	0.25	0.5	0.75
10	0.8780	0.8526	0.8438
20	0.8731	0.8658	0.8341
30	0.8780	0.8682	0.8341
40	0.8804	0.8587	0.8365
50	**0.9016**	0.8574	0.8352
60	0.9001	0.8568	0.8349
70	0.8990	0.8557	0.8345
80	0.8878	0.8547	0.8345
90	0.8754	0.8539	0.8339
100	0.8750	0.8525	0.8324

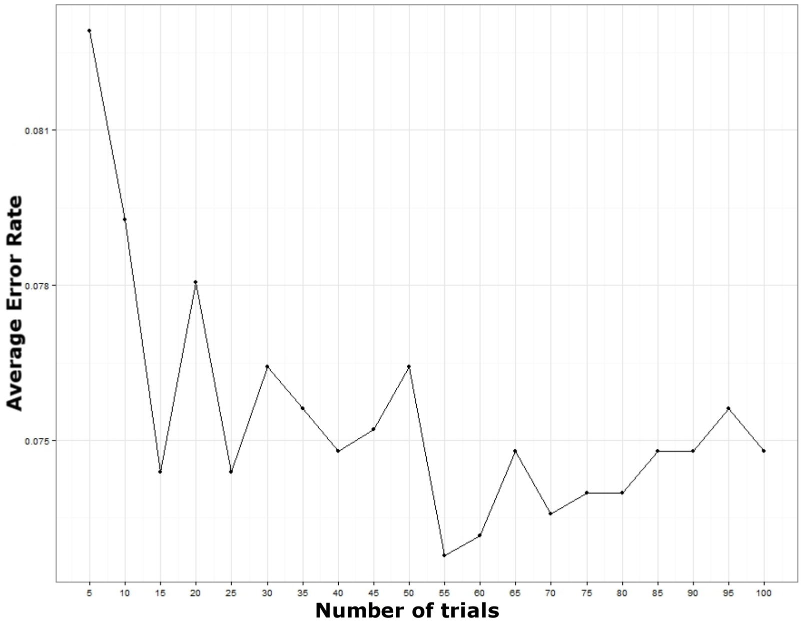

Figure 2: Number of trials optimization in C5.0.

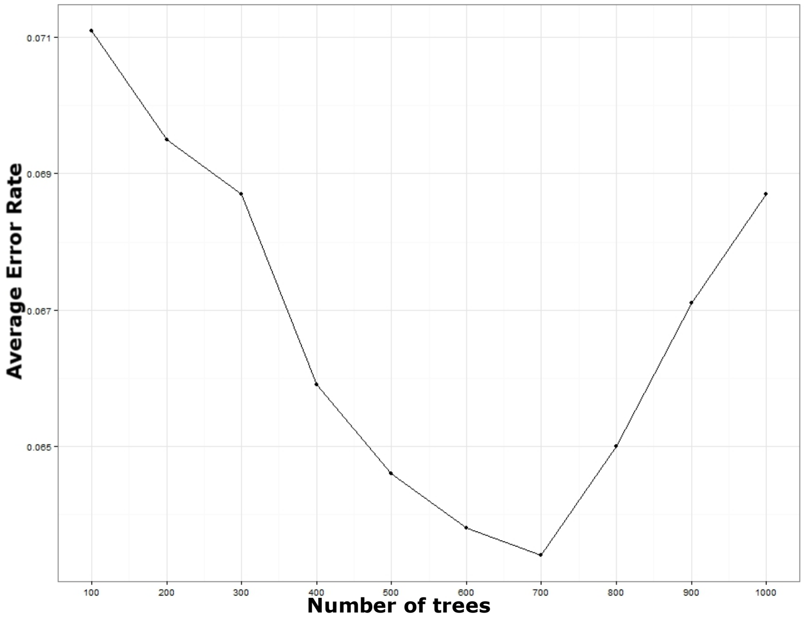

Figure 3: Number of trees optimization in Random forest.

2. Results

Table 7: Average accuracy of single classifiers and ensemble methods across 30 runs in four GBS subtype classification. Ensemble methods are shown in bold.

Method	Average accuracy
**Random forest**	**0.9366**
**C5.0**	**0.9272**
kNN	0.9268
**Boosting**	**0.9195**
SVMLap	0.9163
SVMPoly	0.9142
SVMGaus	0.9126
C4.5	0.9114
SVMLin	0.9069
**Random subspace**	**0.9016**
Naive Bayes	0.9008
**Bagging**	**0.8980**
MLP	0.8915
RBF-DDA	0.8882
SLP	0.8850
JRip	0.8837
LDA	0.8825
MLR	0.8793
OneR	0.7972

Table 8: Impact analysis of the 16 relevant features in the diagnostic model using single classifiers. Highest values are shown in bold.

Single classifier	Using 16 relevant features	Using all features	Difference
	**Average Accuracy**		
KNN	**0.9268**	0.8843	0.0425
SVMLap	0.9163	0.8878	0.0285
SVMPoly	0.9142	0.8692	0.0450
SVMGaus	0.9126	**0.8932**	0.0194
C4.5	0.9114	0.8826	0.0288
SVMLin	0.9069	0.8919	0.0150
Naive Bayes	0.9008	0.8607	0.0401
MLP	0.8915	0.7317	0.1598
RBF-DDA	0.8882	0.6731	**0.2151**
SLP	0.885	0.8207	0.0643
JRip	0.8837	0.8607	0.0230
LDA	0.8825	0.8268	0.0557
MLR	0.8793	0.7602	0.1191
OneR	0.7972	0.7606	0.0366

Table 9: Impact analysis of the 16 relevant features in the diagnostic model using ensemble methods. Highest values are shown in bold.

Ensemble method	Using 16 relevant features	Using all features	Difference
	**Average Accuracy**		
Random forest	**0.9366**	0.9163	0.0203
C5.0	0.9272	0.9150	0.0122
Boosting	0.9195	**0.9187**	0.0008
Random subspace	0.9016	0.7163	**0.1853**
Bagging	0.8980	0.8979	0.0001

Table 10: Average results of ensemble methods across 30 runs in AIDP vs. ALL classification.

Method	Balanced Accuracy	Accuracy	Sensitivity	Specificity	Kappa	AUC
**C5.0**	**0.8171**	**0.9048**	**0.6944**	**0.9398**	**0.6266**	**0.8171**
	0.0976	0.0556	0.1861	0.0565	0.1806	0.0976
**Boosting**	**0.8069**	**0.8992**	**0.6907**	**0.9231**	**0.6046**	**0.8069**
	0.0827	0.0479	0.1750	0.0732	0.1670	0.0827
**Random forest**	**0.7769**	**0.9270**	**0.5667**	**0.9870**	**0.6407**	**0.7769**
	0.0888	0.0306	0.1836	0.0289	0.1645	0.0888
**Bagging**	**0.7681**	**0.8960**	**0.6093**	**0.9269**	**0.5629**	**0.7681**
	0.0818	0.0466	0.1829	0.1091	0.1682	0.0818
**Random subspace**	**0.6815**	**0.9056**	**0.3666**	**0.9953**	**0.4661**	**0.6810**
	0.1240	0.0351	0.2453	0.0129	0.2408	0.1218

Table 11: Balanced accuracy of single classifiers and ensemble methods across 30 runs in AIDP vs. ALL classification. Ensemble methods are shown in bold.

Method	Balanced Accuracy
kNN	0.8227
**C5.0**	**0.8171**
**Boosting**	**0.8069**
C4.5	0.8042
MLP	0.8014
SVMLap	0.8009
**Random forest**	**0.7769**
**Bagging**	**0.7681**
RBF-DDA	0.7648
SLP	0.7551
SVMLin	0.7542
LDA	0.7537
SVMGaus	0.7491
JRip	0.7407
SVMPoly	0.7389
Naive Bayes	0.7231
BLR	0.7181
**Random subspace**	**0.6815**
OneR	0.6222

Table 12: Average results of ensemble methods across 30 runs in AMAN vs. ALL classification.

Method	Balanced Accuracy	Accuracy	Sensitivity	Specificity	Kappa	AUC
**Boosting**	**0.9214**	**0.9341**	**0.8989**	**0.9439**	**0.8384**	**0.9214**
	0.0599	0.0441	0.1045	0.0591	0.1098	0.0599
**Random subspace**	**0.9138**	**0.9143**	**0.9112**	**0.9155**	**0.7992**	**0.9133**
	0.0449	0.0418	0.0788	0.0545	0.0926	0.0442
**C5.0**	**0.9136**	**0.9302**	**0.8750**	**0.9522**	**0.8290**	**0.9136**
	0.0487	0.0385	0.0948	0.0461	0.0911	0.0487
**Random forest**	**0.9033**	**0.9214**	**0.8611**	**0.9456**	**0.8069**	**0.9033**
	0.0550	0.0406	0.1057	0.0424	0.1006	0.0550
**Bagging**	**0.8542**	**0.8952**	**0.7872**	**0.9211**	**0.7303**	**0.8542**
	0.0727	0.0462	0.1624	0.0929	0.1313	0.0727

Table 13: Balanced accuracy of single classifiers and ensemble methods across 30 runs in AMAN vs. ALL classification. Ensemble methods are shown in bold.

Method	Balanced Accuracy
kNN	0.9356
SVMGaus	0.9256
**Boosting**	**0.9214**
SVMPoly	0.9203
SVMLap	0.9181
**Random subspace**	**0.9138**
**C5.0**	**0.9136**
C4.5	0.9067
**Random forest**	**0.9033**
MLP	0.8978
SVMLin	0.8969
SLP	0.8778
Naive Bayes	0.8700
RBF-DDA	0.8689
LDA	0.8594
**Bagging**	**0.8542**
JRip	0.8472
BLR	0.8214
OneR	0.6336

Table 14: Average results of ensemble methods across 30 runs in AMSAN vs. ALL classification.

Method	Balanced Accuracy	Accuracy	Sensitivity	Specificity	Kappa	AUC
**Random forest**	**0.8924**	**0.8960**	**0.8544**	**0.9304**	**0.7889**	**0.8924**
	0.0429	0.0417	0.0814	0.0621	0.0849	0.0429
**Boosting**	**0.8786**	**0.8810**	**0.8671**	**0.8902**	**0.7591**	**0.8786**
	0.0386	0.0375	0.0663	0.0796	0.0762	0.0386
**C5.0**	**0.8782**	**0.8810**	**0.8491**	**0.9072**	**0.7588**	**0.8782**
	0.0494	0.0480	0.0779	0.0532	0.0978	0.0494
**Random subspace**	**0.8688**	**0.8722**	**0.8617**	**0.8798**	**0.7425**	**0.8708**
	0.0410	0.0440	0.0550	0.0664	0.0878	0.0416
**Bagging**	**0.8555**	**0.8587**	**0.8365**	**0.8744**	**0.7137**	**0.8555**
	0.0526	0.0515	0.0960	0.1012	0.1047	0.0526

Table 15: Balanced accuracy of single classifiers and ensemble methods across 30 runs in AMSAN vs. ALL classification. Ensemble methods are shown in bold.

Method	Balanced accuracy
kNN	0.8953
**Random forest**	**0.8924**
C4.5	0.8852
**Boosting**	**0.8786**
**C5.0**	**0.8782**
SVMLap	0.8756
RBF-DDA	0.8695
**Random subspace**	**0.8688**
SLP	0.8644
**Bagging**	**0.8555**
SVMPoly	0.8504
SVMGaus	0.8492
MLP	0.8490
JRip	0.8449
OneR	0.8219
Naive Bayes	0.8166
SVMLin	0.8101
LDA	0.8072
BLR	0.7971

Table 16: Average results of ensemble methods across 30 train-test runs in MF vs. ALL classification.

Method	Balanced Accuracy	Accuracy	Sensitivity	Specificity	Kappa	AUC
**Random subspace**	**0.8471**	**0.9333**	**0.7500**	**0.9525**	**0.6413**	**0.8513**
	0.1067	0.0296	0.2274	0.0349	0.1509	0.1074
**Bagging**	**0.8342**	**0.9024**	**0.7675**	**0.9009**	**0.5498**	**0.8342**
	0.1298	0.0513	0.2460	0.1297	0.2213	0.1298
**C5.0**	**0.8048**	**0.9167**	**0.6667**	**0.9430**	**0.5601**	**0.8048**
	0.1329	0.0487	0.2653	0.0484	0.2145	0.1329
**Boosting**	**0.7572**	**0.9183**	**0.5697**	**0.9447**	**0.5022**	**0.7572**
	0.1346	0.0323	0.2802	0.0881	0.2085	0.1346
**Random forest**	**0.7463**	**0.9254**	**0.5250**	**0.9675**	**0.4973**	**0.7463**
	0.1519	0.0311	0.3104	0.0246	0.2599	0.1519

Table 17: Balanced accuracy of single classifiers and ensemble methods across 30 train-test runs in MF vs. ALL classification. Ensemble methods are shown in bold.

Method	Balanced Accuracy
Naive Bayes	0.8939
**Random subspace**	**0.8471**
**Bagging**	**0.8342**
**C5.0**	**0.8048**
LDA	0.7818
C4.5	0.7691
**Boosting**	**0.7572**
JRip	0.7678
SVMGaus	0.7581
**Random forest**	**0.7463**
BLR	0.7316
SVMLin	0.7221
MLP	0.7193
SVMPoly	0.7086
SLP	0.6875
OneR	0.6853
SVMLap	0.6664
kNN	0.6539
RBF-DDA	0.5042

Table 18: Average results of ensemble methods across 30 runs in AIDP vs. AMAN classification. Standard deviation of each metric is shown in normal font.

Method	Balanced Accuracy	Accuracy	Sensitivity	Specificity	Kappa	AUC
**Bagging**	**0.9597**	**0.9630**	**0.9472**	**0.9722**	**0.9171**	**0.9597**
	0.0458	0.0395	0.0861	0.0551	0.0882	0.0458
**C5.0**	**0.9597**	**0.9611**	**0.9556**	**0.9639**	**0.9139**	**0.9597**
	0.0530	0.0487	0.0868	0.0566	0.1078	0.0530
**Boosting**	**0.9486**	**0.9556**	**0.9278**	**0.9694**	**0.8997**	**0.9486**
	0.0473	0.0397	0.0895	0.0599	0.0889	0.0473
**Random subspace**	**0.9325**	**0.9444**	**0.8833**	**0.9751**	**0.8683**	**0.9292**
	0.0887	0.0652	0.1703	0.0445	0.1589	0.0890
**Random forest**	**0.9097**	**0.9315**	**0.8444**	**0.9750**	**0.8368**	**0.9097**
	0.0805	0.0540	0.1747	0.0497	0.1348	0.0805

Table 19: Balanced accuracy of single classifiers and ensemble methods across 30 runs in AIDP vs. AMAN classification. Ensemble methods are shown in bold.

**Method**	**Balanced Accuracy**
JRip	0.9639
**Bagging**	**0.9597**
**C5.0**	**0.9597**
OneR	0.9583
**Boosting**	**0.9486**
C4.5	0.9458
SVMLin	0.9347
SLP	0.9333
MLP	0.9333
**Random subspace**	**0.9325**
Naive Bayes	0.9278
SVMLap	0.9125
**Random forest**	**0.9097**
SVMGaus	0.9014
RBF-DDA	0.8958
SVMPoly	0.8722
kNN	0.8639
BLR	0.8542
LDA	0.8389

Table 20: Average results of ensemble methods across 30 runs in AIDP vs. AMSAN classification. Standard deviation of each metric is shown in normal font.

**Method**	**Balanced Accuracy**	**Accuracy**	**Sensitivity**	**Specificity**	**Kappa**	**AUC**
**Random forest**	**0.9102**	**0.9387**	**0.8556**	**0.9649**	**0.8312**	**0.9102**
	0.0588	0.0430	0.1136	0.0485	0.1137	0.0588
**Boosting**	**0.8972**	**0.9160**	**0.8532**	**0.9412**	**0.7797**	**0.8972**
	0.0609	0.0539	0.1032	0.0623	0.1308	0.0609
**Bagging**	**0.8854**	**0.9067**	**0.8480**	**0.9228**	**0.7565**	**0.8854**
	0.0774	0.0675	0.1211	0.0798	0.1635	0.0774
**C5.0**	**0.8683**	**0.8893**	**0.8278**	**0.9088**	**0.7095**	**0.8683**
	0.0755	0.0563	0.1348	0.0569	0.1434	0.0755
**Random subspace**	**0.8674**	**0.9093**	**0.7834**	**0.9491**	**0.7459**	**0.8662**
	0.0755	0.0481	0.1524	0.0544	0.1324	0.0744

Table 21: Balanced accuracy of single classifiers and ensemble methods across 30 runs in AIDP vs. AMSAN classification. Ensemble methods are shown in bold.

Method	Balanced Accuracy
**Random forest**	**0.9102**
SVMLap	0.9061
SVMPoly	0.8990
**Boosting**	**0.8972**
MLP	0.8949
SVMGaus	0.8931
SVMLin	0.8928
kNN	0.8899
**Bagging**	**0.8854**
SLP	0.8841
Naive Bayes	0.8728
C4.5	0.8692
**C5.0**	**0.8683**
**Random subspace**	**0.8674**
JRip	0.8588
BLR	0.8423
RBF-DDA	0.8339
OneR	0.8316
LDA	0.7836

Table 22: Average results of ensemble methods across 30 runs in AIDP vs. MF classification. Standard deviation of each metric is shown in normal font.

Method	Balanced Accuracy	Accuracy	Sensitivity	Specificity	Kappa	AUC
**Random subspace**	**0.8951**	**0.8733**	**0.7944**	**0.9917**	**0.7527**	**0.8930**
	0.0673	0.0785	0.1288	0.0456	0.1494	0.0671
**Bagging**	**0.8750**	**0.8533**	**0.8333**	**0.9167**	**0.7158**	**0.8806**
	0.1089	0.1167	0.1578	0.1681	0.2223	0.0867
**Random forest**	**0.8306**	**0.8267**	**0.8111**	**0.8500**	**0.6460**	**0.8306**
	0.0847	0.0785	0.1366	0.1925	0.1626	0.0847
**C5.0**	**0.8014**	**0.7967**	**0.7778**	**0.8250**	**0.5820**	**0.8014**
	0.1167	0.0999	0.1337	0.2555	0.2221	0.1167
**Adaboost**	**0.7694**	**0.7767**	**0.7167**	**0.8222**	**0.5333**	**0.7806**
	0.1275	0.1165	0.2037	0.2303	0.2337	0.0996

Table 23: Balanced accuracy of single classifiers and ensemble methods across 30 runs in AIDP vs. MF classification. Ensemble methods are shown in bold.

Method	Balanced Accuracy
**Random subspace**	**0.8951**
**Bagging**	**0.8750**
OneR	0.8667
JRip	0.8528
kNN	0.8417
MLP	0.8347
Naive Bayes	0.8319
**Random forest**	**0.8306**
C4.5	0.8236
SVMGaus	0.8222
SVMPoly	0.8069
SVMLap	0.8028
**C5.0**	**0.8014**
SLP	0.7708
**Boosting**	**0.7694**
SVMLin	0.7542
RBF-DDA	0.7042
LDA	0.6250
BLR	0.6111

Table 24: Average results of ensemble methods across 30 runs in AMAN vs. AMSAN classification. Standard deviation of each metric is shown in normal font.

Method	Balanced Accuracy	Accuracy	Sensitivity	Specificity	Kappa	AUC
**Random forest**	**0.9474**	**0.9505**	**0.9333**	**0.9614**	**0.8950**	**0.9474**
	0.0415	0.0347	0.0859	0.0389	0.0752	0.0415
**Random subspace**	**0.9390**	**0.9473**	**0.9057**	**0.9736**	**0.8872**	**0.9396**
	0.0420	0.0344	0.0840	0.0359	0.0752	0.0414
**Boosting**	**0.9284**	**0.9355**	**0.9216**	**0.9352**	**0.8625**	**0.9284**
	0.0464	0.0397	0.0689	0.0960	0.0865	0.0464
**C5.0**	**0.9242**	**0.9290**	**0.9028**	**0.9456**	**0.8505**	**0.9242**
	0.0651	0.0625	0.0981	0.0725	0.1293	0.0651
**Bagging**	**0.8940**	**0.8989**	**0.8877**	**0.9003**	**0.7879**	**0.8940**
	0.0648	0.0632	0.0768	0.1065	0.1327	0.0648

Table 25: Balanced accuracy of single classifiers and ensemble methods across 30 runs in AMAN vs. AMSAN classification. Ensemble methods are shown in bold.

**Method**	**Balanced accuracy**
kNN	0.9588
**Random forest**	**0.9474**
SVMLap	0.9406
**Random subspace**	**0.9390**
MLP	0.9344
**Boosting**	**0.9284**
RBF-DDA	0.9265
**C5.0**	**0.9242**
SVMGaus	0.9236
SLP	0.9202
SVMPoly	0.9189
C4.5	0.9138
SVMLin	0.9125
Naive Bayes	0.9091
**Bagging**	**0.8940**
LDA	0.8838
JRip	0.8827
BLR	0.8559
OneR	0.8437

Table 26: Average results of ensemble methods across 30 runs in AMAN vs. MF classification. Standard deviation of each metric is shown in normal font.

**Method**	**Balanced Accuracy**	**Accuracy**	**Sensitivity**	**Specificity**	**Kappa**	**AUC**
**Random forest**	**0.9333**	**0.9625**	**0.9917**	**0.8750**	**0.8908**	**0.9333**
	0.0838	0.0452	0.0335	0.1706	0.1346	0.0838
**Bagging**	**0.9181**	**0.9313**	**0.9611**	**0.8750**	**0.8214**	**0.9181**
	0.0705	0.0502	0.0811	0.1431	0.1271	0.0705
**Boosting**	**0.9167**	**0.9417**	**0.9639**	**0.8694**	**0.8449**	**0.9201**
	0.0922	0.0655	0.0779	0.1662	0.1888	0.0967
**C5.0**	**0.9069**	**0.9313**	**0.9556**	**0.8583**	**0.8201**	**0.9069**
	0.0802	0.0664	0.0750	0.1421	0.1619	0.0802
**Random subspace**	**0.7543**	**0.8708**	**0.9917**	**0.5083**	**0.5749**	**0.7500**
	0.1227	0.0567	0.0253	0.2583	0.2123	0.1228

Table 27: Balanced accuracy of single classifiers and ensemble methods across 30 in AMAN vs. MF classification. Ensemble methods are shown in bold.

Method	Balanced Accuracy
SVMGaus	0.9542
SVMLap	0.9542
RBF-DDA	0.9528
Naive Bayes	0.9486
SVMLin	0.9431
kNN	0.9417
SVMPoly	0.9375
**Random forest**	**0.9333**
**Bagging**	**0.9181**
**Boosting**	**0.9167**
MLP	0.9125
**C5.0**	**0.9069**
SLP	0.9056
LDA	0.9056
C4.5	0.8889
BLR	0.8833
oneR	0.8806
JRip	0.8792
**Random subspace**	**0.7543**

Table 28: Average results of ensemble methods across 30 runs in AMSAN vs. MF classification. Standard deviation of each metric is shown in normal font.

Method	Balanced Accuracy	Accuracy	Sensitivity	Specificity	Kappa	AUC
**Random subspace**	**0.8879**	**0.8928**	**0.8964**	**0.8750**	**0.6786**	**0.8857**
	0.0862	0.0556	0.0561	0.1574	0.1590	0.0855
**C5.0**	**0.8651**	**0.9130**	**0.9386**	**0.7917**	**0.7032**	**0.8651**
	0.0898	0.0379	0.0480	0.1979	0.1272	0.0898
**Bagging**	**0.8645**	**0.8957**	**0.8961**	**0.8329**	**0.7195**	**0.8978**
	0.1789	0.1749	0.1869	0.2177	0.2482	0.0757
**Random forest**	**0.8539**	**0.9217**	**0.9579**	**0.7500**	**0.7113**	**0.8539**
	0.1061	0.0418	0.0350	0.2177	0.1727	0.1061
**Boosting**	**0.8246**	**0.9058**	**0.9364**	**0.7127**	**0.6519**	**0.8246**
	0.1155	0.0458	0.0717	0.2463	0.1888	0.1155

Table 29: Balanced accuracy of single classifiers and ensemble methods across 30 runs in AMSAN vs. MF classification. Ensemble methods are shown in bold.

**Method**	**Balanced Accuracy**
Naive Bayes	0.8980
**Random subspace**	**0.8879**
LDA	0.8656
**C5.0**	**0.8651**
**Bagging**	**0.8645**
MLP	0.8588
**Random forest**	**0.8539**
SLP	0.8493
SVMGaus	0.8480
SVMLap	0.8316
JRip	0.8248
**Boosting**	**0.8246**
kNN	0.8099
BLR	0.8042
C4.5	0.8026
SVMLin	0.8013
SVMPoly	0.7974
OneR	0.7811
RBF-DDA	0.6757
